# Downregulating DNA methyltransferase 3B by suppressing the PI3K/Akt signaling pathway enhances the chemosensitivity of glioblastoma to temozolomide

**DOI:** 10.1007/s12035-024-04041-7

**Published:** 2024-02-17

**Authors:** Wenwu Kan, Linhui Gao, Jingnan Chen, Li Chen, Guojun Zhang, Bilie Hao, Min He, Xudong Chen, Cheng Wang

**Affiliations:** 1 Department of Neurosurgery, The Second Affiliated Hospital of Zhejiang Chinese Medical University, Hangzhou, 310011 Zhejiang China; 2 The First Clinical Medical College of Zhejiang, Chinese Medical University, Hangzhou, 310053 Zhejiang China

**Keywords:** Glioblastoma, Temozolomide, DNMT3B, Chemoresistance

## Abstract

Glioblastoma (GBM) is the most common malignant brain tumor and has the poorest prognosis attributed to its chemoresistance to temozolomide (TMZ), the first-line drug for treating GBM. TMZ resistance represents a significant obstacle to successful GBM treatment, necessitating the development of new strategies to overcome this resistance and augment the chemosensitivity of GBM cells to TMZ. This study established a TMZ-resistant U251 (U251-TMZ) cell line by exposing it to increasing doses of TMZ in vitro. We focused on the DNA methyltransferase 3B (DNMT3B) gene, phosphorylated Akt (p-Akt), total Akt (t-Akt), phosphorylated PI3K (p-PI3K), and total PI3K (t-PI3K) protein expression. Results showed that the DNMT3B gene was significantly upregulated in the U251-TMZ cell line. The p-Akt and p-PI3K protein expression in U251-TMZ cells was also significantly elevated. Moreover, we found that DNMT3B downregulation was correlated with the increased chemosensitivity of GBM cells to TMZ. LY294002 suppressed the PI3K/Akt signaling pathway, leading to a notable inhibition of PI3K phosphorylation and a significant decrease in DNMT3B expression in U251-TMZ cells. Given that DNMT3B expression is mediated by the PI3K/Akt signaling pathway, its downregulation further increased the chemosensitivity of GBM cells to TMZ and therefore is a promising therapeutic for GBM treatment. Our results suggested that DNMT3B downregulation can inhibit the proliferation of GBM cells and induce GBM cell apoptosis in vitro. In addition, the PI3K/Akt signaling pathway plays an important role in the chemosensitivity of GBM cells to TMZ by regulating DNMT3B expression.

## Introduction

Gliomas are aggressive, infiltrative and malignant tumors that originate from the neuroectoderm and account for approximately 81% of all primary malignant brain tumors [1, 2]. Glioblastoma (GBM), the most aggressive type of gliomas, is notorious for its infiltrative behavior and recalcitrance to treatment, conferring patients a dismal median survival of 12–15 months and a 5-year survival rate below 5% [1, 3]. The current standard treatments for GBM primarily comprise surgical resection combined with postoperative radiotherapy, chemotherapy, and tumor-treating fields [4–6]. Although radiation therapy has the potential to eliminate radiosensitive GBM cells, chemotherapy remains a crucial component of treatment following surgical intervention. Temozolomide (TMZ), a DNA-alkylating, highly efficacious chemical agent, is commonly employed in the management of primary malignant brain tumors by inducing lethal DNA lesions in fast-dividing tumor cells [7–9]. However, TMZ resistance represents a significant obstacle to successful GBM treatment, necessitating the development of strategies to overcome this resistance and augment the chemosensitivity of GBM cells to TMZ.

Modifications to DNA methylation are significant contributors to the pathogenesis and progression of various tumors, including GBM [10]. Aberrant DNA methylation has also been linked to the development of chemotherapy-resistant tumor phenotypes [11]. Epigenetic modification, including chromatin remodeling, histone modification, and DNA methylation, is a biological process that regulates gene expression without altering DNA sequences [12, 13] and is a well-established key factor in tumors [10]. DNA methylation occurring at the fifth position of cytosines at the CpG site is catalyzed by DNA methyltransferases (DNMTs) [14, 15]. Several DNMTs are mainly involved in DNA methylation, including DNMT1, DNMT3A, and DNMT3B. In particular, DNMT3A and DNMT3B catalyze the de novo methylation of DNA [16]. Studies on tumor cell lines and primary tumor tissues provided compelling evidence that aberrant changes in DNA methylation patterns are a hallmark of cancer [17]. DNMT3B plays a crucial role in embryonic development and aberrant DNA methylation in carcinogenesis [18]. Inhibiting DNMT3B can reduce the degree of methylation of tumor suppressor genes by facilitating demethylation, thereby significantly decreasing the proliferation and invasion ability of tumor cells [19].

Phosphoinositide 3- kinase (PI3K) is an important chemoresistance-causing factor in the treatment of cancer. Protein kinase B (Akt) is an important downstream effector of PI3K signaling that can modulate several pathways, including the stimulation of cell growth, modulation of cellular metabolism, and inhibition of apoptosis. The PI3K/Akt pathway plays a pivotal role in multidrug resistance by synergizing the upstream and downstream targets involved in the modulation of cellular metabolism, cell growth, and apoptosis [20]. In a prior study, Mei [21] demonstrated for the first time that the PI3K/Akt pathway regulates DNMT3B expression at the transcriptional and posttranscriptional levels in human hepatocellular carcinoma lines. The PI3K/Akt pathway participates in the mediation of DNMT3B expression in some cancers, but its role in TMZ resistance in GBM cells has rarely been reported.

We hypothesized that DNMT3B overexpression can make GBM cells resistant to TMZ-induced cell death. Considering that the PI3K/Akt pathway participates in chemoresistance and mediates DNMT3B expression, we could reasonably postulate that DNMT3B inhibition plays a tumor suppressor role in GBM in a PI3K/Akt pathway-dependent manner. Therefore, this study assessed whether the PI3K/Akt pathway regulates TMZ resistance in GBM cells through DNMT3B expression and upstream and downstream biomolecules. The findings may clarify the mechanism of TMZ resistance in GBM cells and provide promising therapeutic options for GBM treatment.

## Material and methods

### Cell lines and cell culture

The human GBM-derived cancer line U251 was purchased from the Cell Bank Type Culture Collection of the Chinese Academy of Sciences (Shanghai, China). The cell line was cultured at 37 °C under a humidified atmosphere of 5% CO_2_ by using Dulbecco’s modified Eagle’s medium (DMEM, Gibco, Carlsbad, CA, USA) supplemented with 10% fetal bovine serum (FBS), 2 mM glutamine, 100 U/mL penicillin, and 100 µg/mL streptomycin (Sigma, St. Louis, MO, USA).

### Stepwise induction of drug‑resistant strains

A GBM cell line with acquired resistance to TMZ was developed from the U251 cell line. First, the U251 cell line was cultured in six-well plates. Following overnight incubation at 37 °C, the medium was replaced with a fresh one containing varying TMZ concentrations ranging from 50 µM to 1600 µM for further induction. TMZ treatment was repeated every 24 h for 5 consecutive days, and the cells were exposed to fresh TMZ every 3 days for 3 weeks. The surviving colonies and established TMZ-resistant U251 (U251-TMZ) cell lines were selected by MTT assay.

### MTT assay

The cell viability of a human GBM-derived cancer line (U251) was detected using the MTT Cell Proliferation and Cytotoxicity Assay Kit purchased from MedChemExpress (Monmouth Junction, NJ, USA) in accordance with the manufacturer’s instructions. The cell lines (1 × 104 cells/well) were all cultured in 24-well plates and then transfected with Vector/NR5A2 or Scramble/NR5A2-sh2#. The transfected U251 cells were cultured for 12, 24, 48, and 72 h. Afterward, the MTT solution was removed and replaced with 150 μL of 4% dimethyl sulfoxide (DMSO; Sigma). Half-maximal inhibitory concentration (IC50) was detected to determine TMZ cytotoxicity. A microplate reader (Bio-Tek, Instruments, Neufahrn, Germany) was used to measure the absorbance at 490 nm. Experiments were performed in triplicate.

### RNA extraction and reverse transcription-quantitative polymerase chain reaction (PCR)

Total RNA was extracted from the cell lines (U251 and U251-TMZ) using TRIzol reagent (Invitrogen; Thermo Fisher Scientific, Inc.) in accordance with the manufacturer’s protocol. cDNA was synthesized by using a High-Capacity cDNA Reverse Transcription Kit with RNase Inhibitor (Fermentas; Thermo Fisher Scientific, Inc.) and Maxima SYBR Green/ROX qPCR Master Mix (Invitrogen; Thermo Fisher Scientific, Inc.) following the manufacturer’s protocol. qPCR analysis was performed using the QuantStudio 6 Flex Real-Time PCR system (Applied Biosystems; Thermo Fisher Scientific, Inc.). The program was as follows: denaturation at 95 °C for 15 min, followed by 40 cycles of 10 s at 95 °C for annealing and 32 s at 60 °C for extension. The melting curve started at 95 °C for 15 s, followed by 60 °C for 1 min and ended with 15 s at 95 °C. The primers are shown in Table 1 [21]. The 2-^∆∆^CT method was used for the relative quantification of gene expression levels following the quantitative real-time PCR (qPCR) experiments.

**Table 1:** Sequences of Primers used for experiments in this study

Gene (human)	Forward primer	Reversed primer	Amplicon (bp)
DNMT3B (for all isoforms)	GAGTCCATTGCTGTTGGAACCG	ATGTCCCTCTTGTCGCCAACCT	305
GAPDH	AAGGCTGAGAACGGGAAGC	GAGGGATCTCGCTCCTGGA	68

### Construction of DNMT3B RNAi lentiviral vectors

On the basis of the gene sequence of DNMT3B in GenBank (Gene ID: 1789), primers for DNMT3B siRNA and negative control were designed and synthesized by GenePharma (Shanghai, China). The interference primers for si-DNMT3B (DNMT3B-siRNA) were as follows: 5ʹ-AACAAGACTCGAAGACGCA-3ʹ. The primers for the interference control siRNA were as follows (5ʹ-TTCTCCGAACGTGTCACGT-3ʹ). The U251-TR cells were then transfected with control-siRNA and DNMT3B-siRNA lentiviral vectors.

### Flow cytometry analyses

For the cell apoptosis assay, 2 × 10^5^ cells were seeded in six-well plates and transfected with control-siRNA and DNMT3B-siRNA lentiviral vectors. Cell apoptosis was detected using an Annexin V-fluorescein isothiocyanate (FITC) kit (Sigma, St. Louis, MO, USA). Double staining with FITC-conjugated annexin V and propidium iodide (PI) was performed as follows. The cells and floating cells were washed twice with 4 °C PBS, resuspended in binding buffer (10 mM HEPES/NaOH, 140 mM NaCl, 2 mM KCl), and harvested after 48 h of transfection. Annexin V was added for 15 min in the dark. The cells were then washed, centrifuged, and resuspended in binding buffer. Before flow cytometric analysis, PI was added to each sample. The annexin V + /PI- cells were early apoptotic cells. Finally, cell apoptosis was detected by a FACS Cano II flow cytometer (Becton Dickinson, Franklin Lakes, NJ, USA).

### Colony formation assay

Following transfection, 1 × 10^3^ U251 cells were plated into six-well plates and incubated at 37 °C for 21 days. A colony was defined as a clump of cells that could be clearly distinguished from another colony after staining. The cell colonies were fixed with 10% formaldehyde for 20 min at room temperature, stained at 37 °C with 0.1% Coomassie Brilliant Blue R250 for 2 min, visualized using a camera, and counted.

### Western blotting

Total protein was extracted from the U251 and U251-TMZ cell lines using RIPA lysis buffer (Beyotime Institute of Biotechnology) and quantified using a bicinchoninic acid assay kit (Beyotime Institute of Biotechnology). Afterward, 20 μg of protein/lane was separated via 10% SDS‒PAGE. The separated proteins were subsequently transferred onto polyvinylidene fluoride membranes (Millipore Sigma) and blocked with 5% nonfat milk in TBS-Tween-20 (TBST; 0.1% Tween-20) at room temperature for 1 h. The membranes were then incubated at 4 °C overnight with primary antibodies, including p-Akt (ab81283, 1:800), t-Akt (ab8805, 1:1000), p-PI3K (ab154598, 1:800), t-PI3K (ab32089, 1:1000), and GAPDH (ab181602, 1:1000). Following the primary antibody incubation step, the membranes were rinsed five times with TBST and incubated with the following secondary antibodies: horseradish peroxidase-conjugated goat anti-rabbit secondary antibody (1:10,000; cat. no. ab205718; Abcam), and horseradish peroxidase-conjugated goat anti-mouse secondary antibody (1:10,000; cat. no. ab205719; Abcam) for 1 h at room temperature. Protein bands were visualized using an enhanced chemiluminescence (Thermo Fisher Scientific, Inc.) reagent, and densitometric analysis was performed using ImageJ version 1.5d software (National Institutes of Health).

### Statistical analysis

All data analyses were carried out using SPSS 22.0 (IBM, Armonk, New York, USA). All quantitative data are presented as the mean ± SEM. Comparison of data between different groups was performed using an independent sample t test. The significance levels of the statistical tests were set at *p < 0.05 and **p < 0.001.

## Results

### Establishment of U251-TMZ cells

**Fig. 1:**
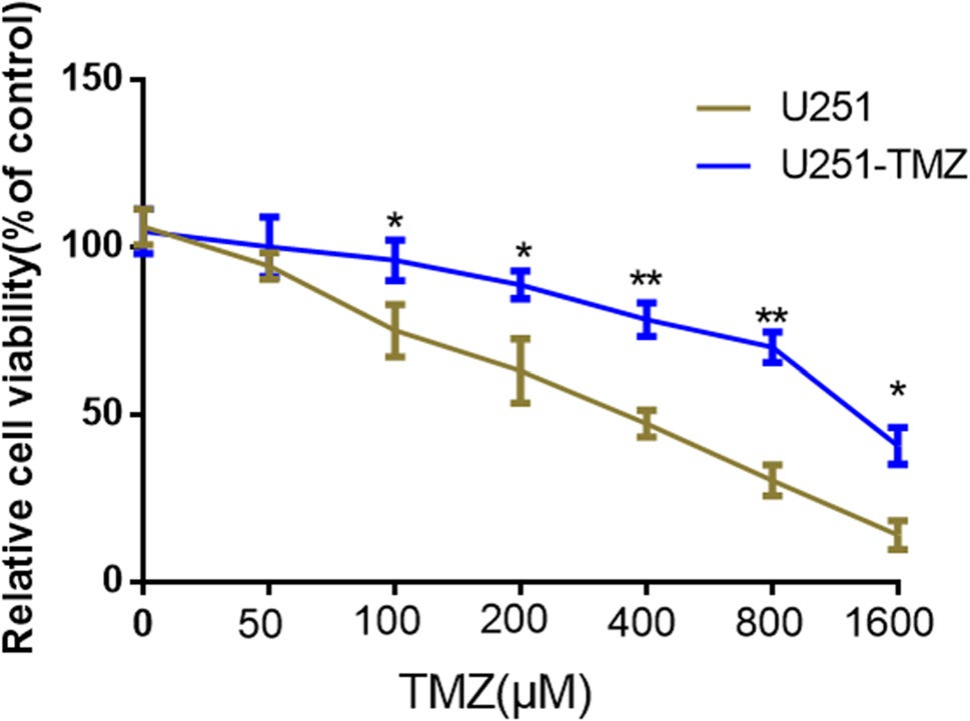
Establishment and validation of temozolomide-resistant U251 cell lines. *p < 0.05, **p < 0.001

We first established U251-TMZ cell lines by exposing cells to increasing TMZ concentrations. U251-TMZ and U251 cells were treated with different concentrations (0, 50, 100, 200, 400, 800, and 1600 µM) of TMZ for 24 h. MTT assays showed that the U251-TMZ cell viability was not affected (compared with that of U251 cells) when the TMZ concentration was ≤ 50 µM within 24 h. When the TMZ concentration was increased to 100 µM, the U251 cell viability significantly decreased compared with that of the U251-TMZ cells. With the increasing TMZ concentrations, the U251 and U251-TMZ cell viability decreased. However, the U251 cell viability significantly decreased compared with that of the U251-TR cells at TMZ concentrations of 100, 200, 400, 800, and 1600 µM (Fig. 1). These results indicated that a TMZ-resistant GBM cell line (U251-TMZ) was successfully established.

### Differentially expressed genes

**Fig. 2:**
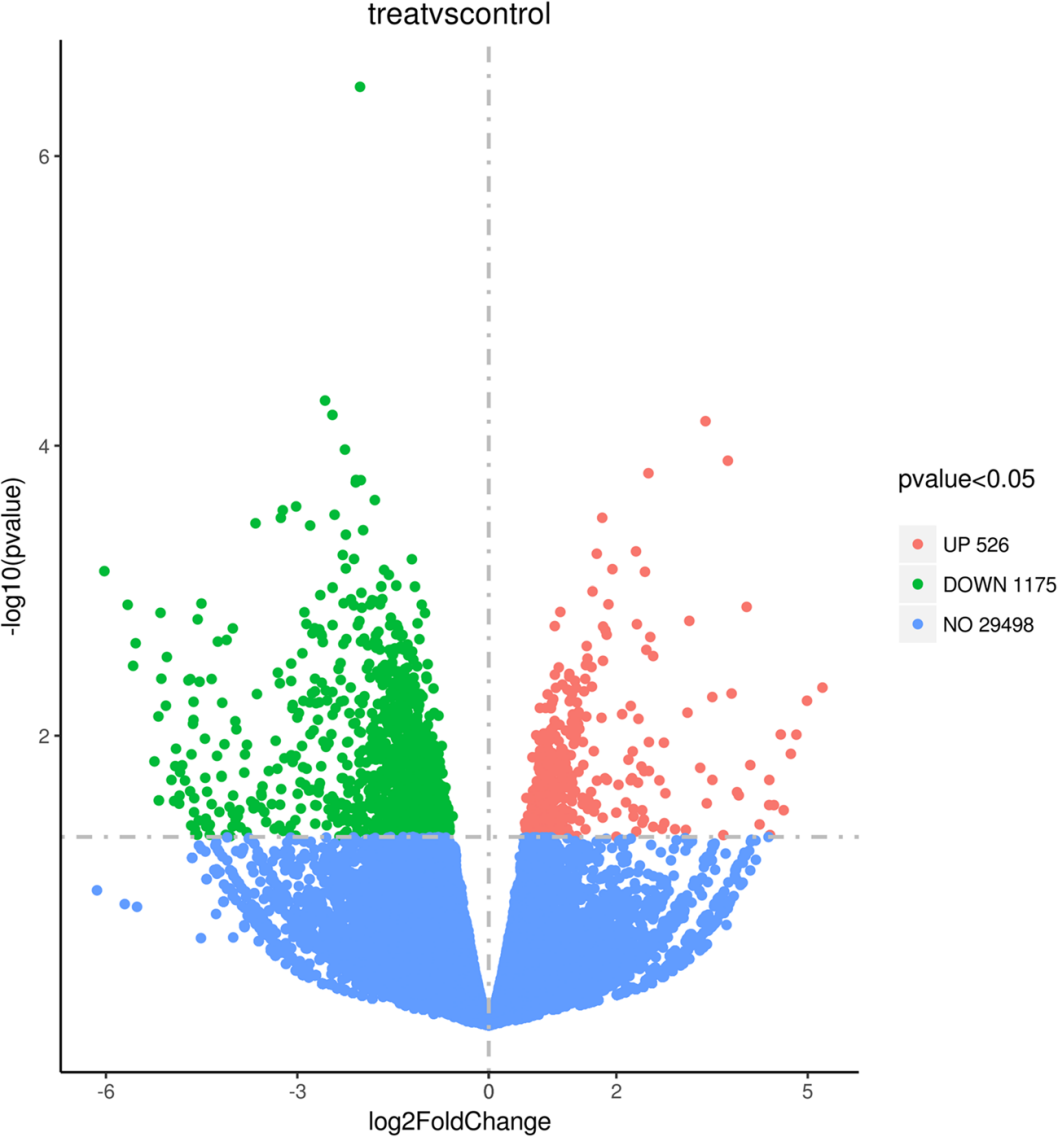
Volcano plot for the analysis results of the differential gene expression of U251 and U251-TMZ cell lines. The screening condition was *p* < 0.05

**Fig. 3:**
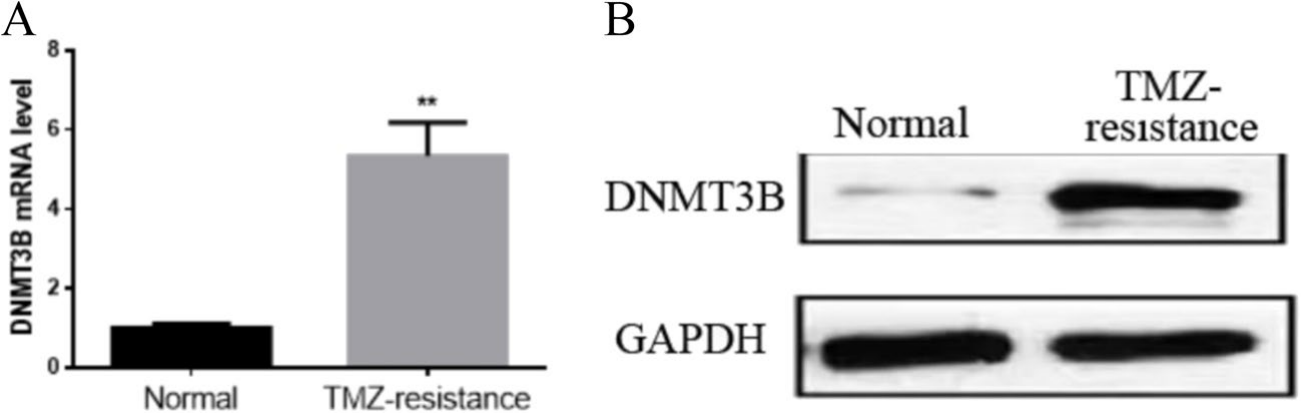
Expression levels of DNMT3B according to qPCR and Western blot analysis. ** p < 0.001

The entire transcriptomes of the U251 and U251-TMZ cell lines were sequenced. As shown in Fig. 2, 29,498 mRNA genes were not altered, 1175 mRNA genes were significantly decreased (*p* < 0.05), and526 mRNA genes were significantly upregulated in the U251-TMZ cell line compared with those in the U251 cell line. The qPCR and Western blot analysis further revealed that the expression levels of DNMT3B gene (Fig. 3A) and DNMT3B protein (Fig. 3B) were significantly upregulated in the U251-TMZ cell line compared with those in the U251 cell line.

### DNMT3B knockout inhibited the viability and colony formation and enhanced the chemosensitivity of GBM cells to TMZ treatment

**Fig. 4:**
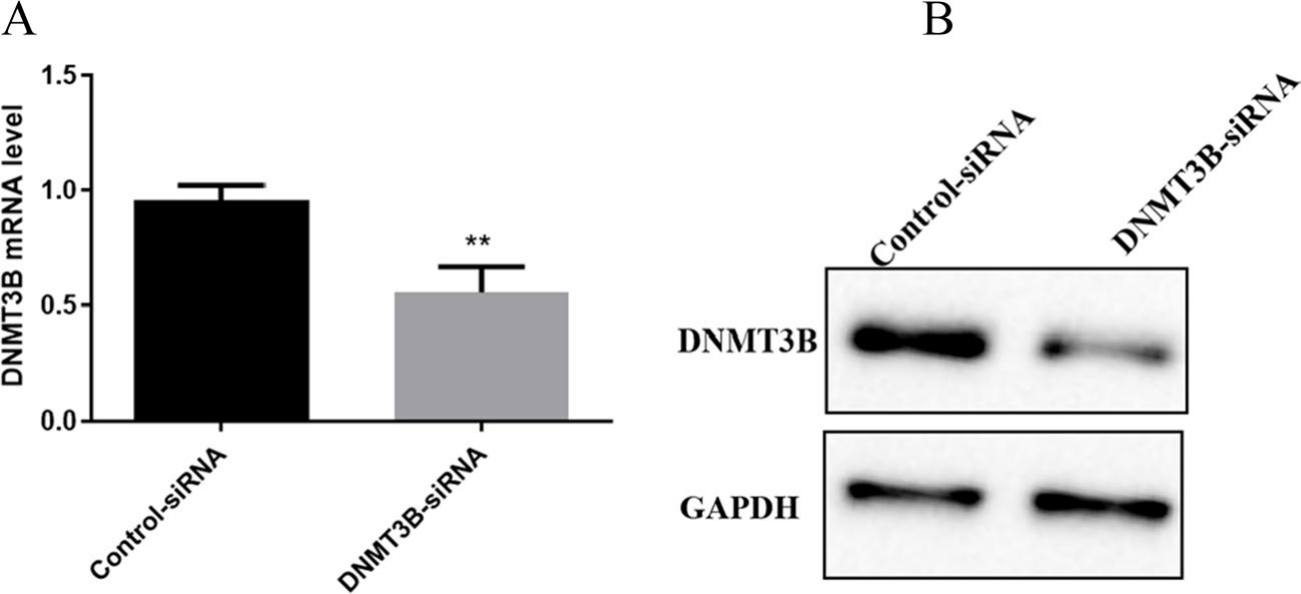
Expression levels of DNMT3B after the transfection of DNMT3B-siRNA lentiviral vectors and control-siRNA according to qPCR (A) and Western blot analysis (B). ** p < 0.001

DNMT3B-siRNA lentiviral vectors and control-siRNA were transfected into the U251-TMZ cell line to investigate the biological function of DNMT3B in GBM cells. The transfection efficiency was evaluated using qPCR and Western blot analysis. The DNMT3B-siRNA lentiviral vector-transfected cells had significantly downregulated DNMT3B expression levels compared with the control siRNA-transfected cells (Figs. 4A and B).

**Fig. 5:**
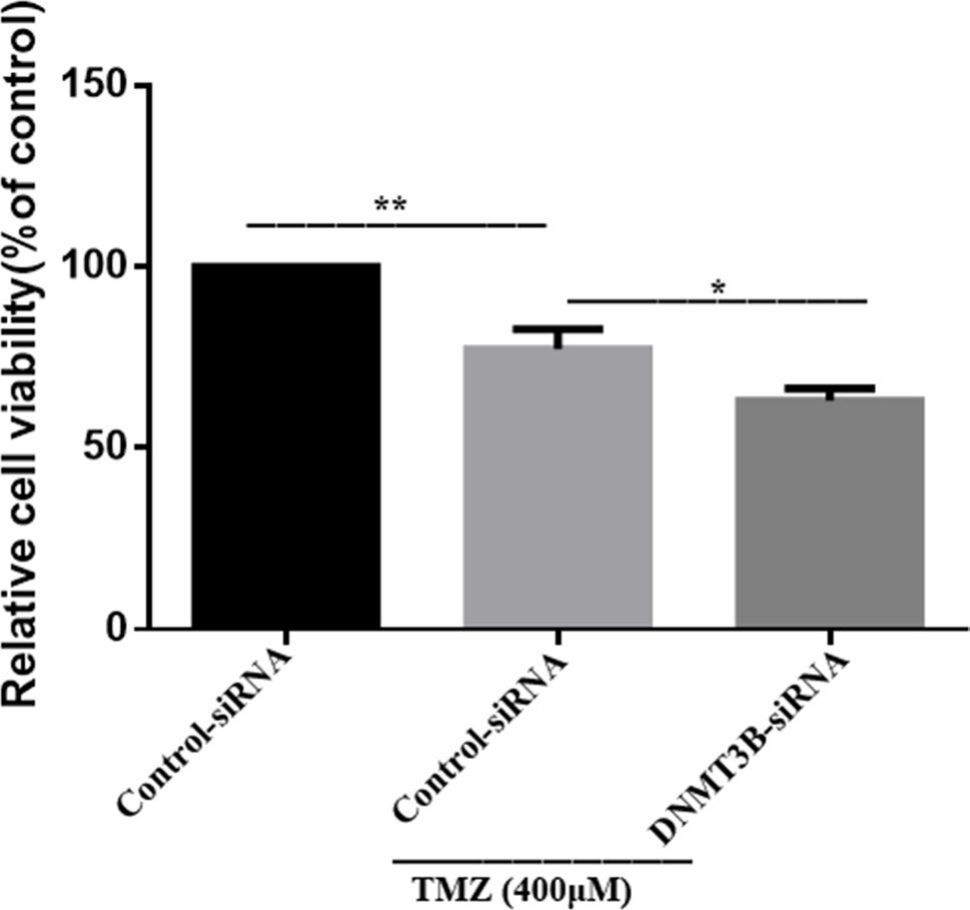
Knockout of DNMT3B enhances the chemosensitivity of glioblastoma cells to TMZ. *p < 0.05, **p < 0.001

**Fig. 6:**
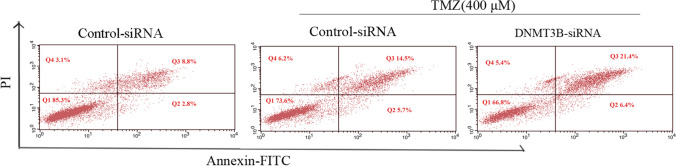
Flow cytometry analyses showed cell apoptosis (DNMT3B-siRNA cells, early apoptosis: 6.4%, late apoptosis: 21.4%; Control-siRNA cells, early apoptosis: 2.5%, late apoptosis: 14.5%). *p < 0.05

**Fig. 7:**
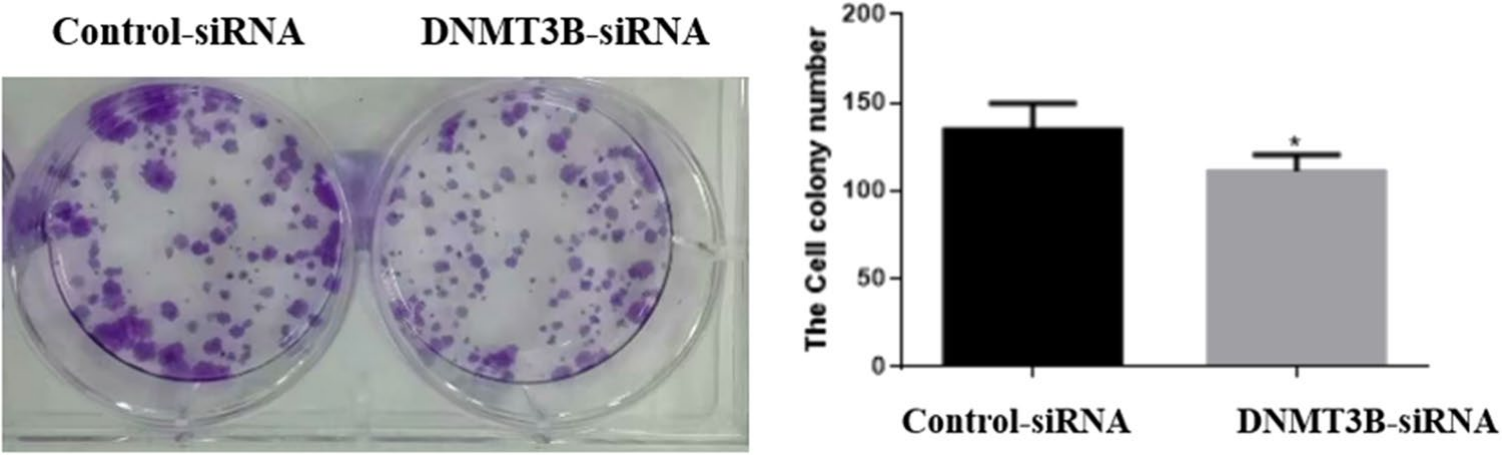
Colony formation assay showed that the number of colonies formed in the U251-TMZ cells transfected with the DNMT3B-siRNA lentiviral vectors significantly decreased compared with that in the control-siRNA-transfected cells. *p < 0.05

Following successful transfection, MTT assay was used to test cell viability after downregulating DNMT3B. The results showed that the DNMT3B knockout cells showed significantly less cell viability following 24 h of incubation with 400 µM TMZ compared with the control siRNA-transfected cells (Fig. 5). To determine whether DNMT3B downregulation regulates chemosensitivity, we measured the apoptosis of DNMT3B knockout cells after treatment with 400 µM TMZ. Flow cytometry analyses showed that the apoptosis rates of DNMT3B-siRNA lentiviral vector-transfected cells significantly increased compared with those of the control siRNA-transfected cells (Fig. 6). Colony formation assay is an effective way to detect the proliferation ability of single cells. We used the clonogenic assay to assess cell proliferative capacity and viability after treatment with TMZ. The results revealed that the number of colonies formed by U251-TMZ cells transfected with the DNMT3B-siRNA lentiviral vectors was significantly lower than that formed by the control-siRNA-transfected cells, suggesting that DNMT3B downregulation can inhibit GBM cell proliferation (Fig. 7).

### The PI3K/Akt signaling pathway regulates DNMT3B protein expression levels, thus controlling the chemosensitivity of GBM cells to TMZ

**Fig. 8:**
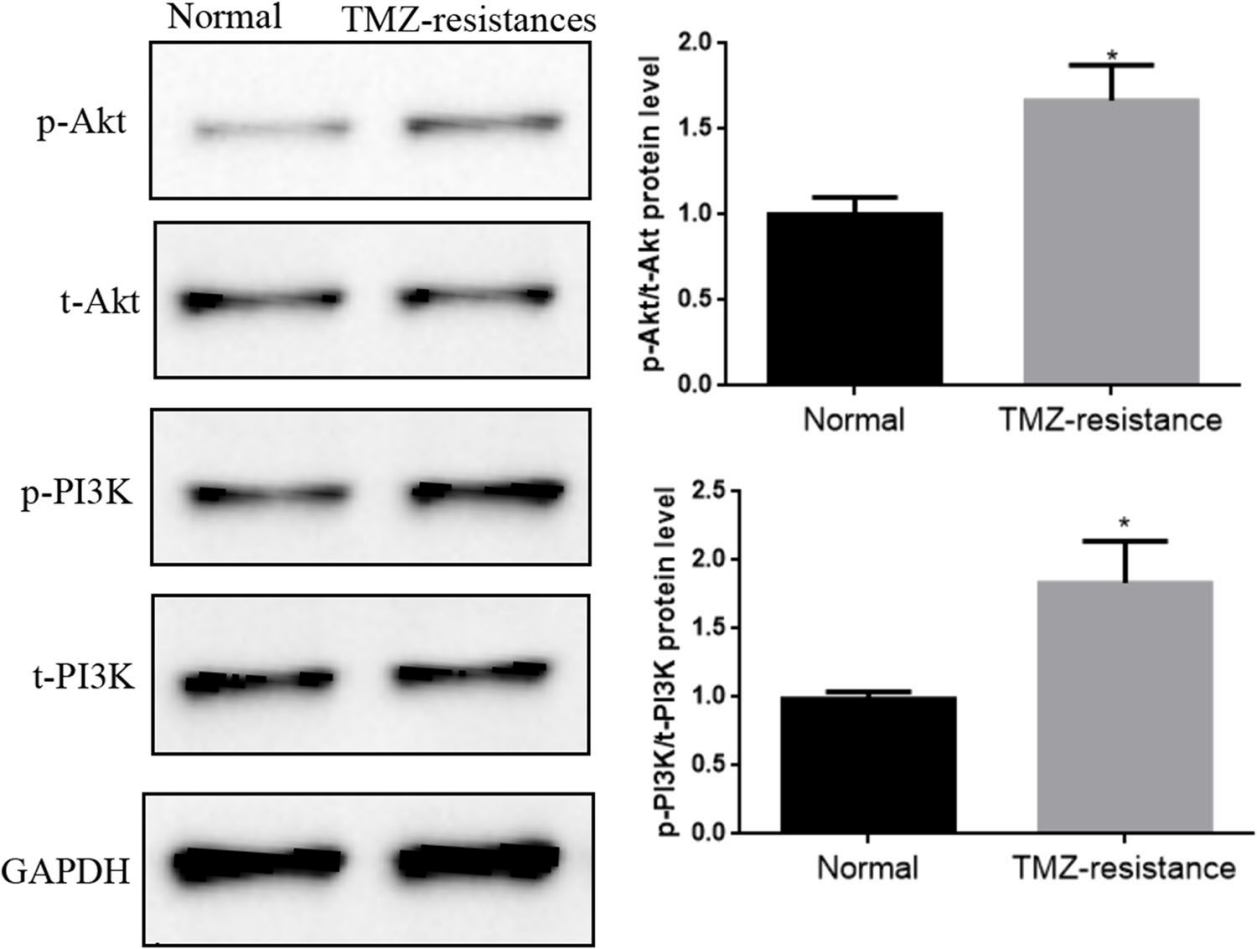
Western blot analysis showed that the p-Akt and p-PI3K protein expression in U251-TMZ cells significantly increased compared with that in U251 cells. *p < 0.05

DNA methylation is a crucial regulator in tumor development[10]. A previous study demonstrated that the PI3K/Akt pathway regulates DNMT3B expression in some tumors[21]. We further measured the expression of PI3K/Akt pathway-related proteins (total PI3K, phosphorylated PI3K, total Akt, and phosphorylated Akt) in U251 cells and U251-TMZ cells. The results showed that phosphorylated Akt (p-Akt) and PI3K (p-PI3K) protein expression in the U251-TMZ cells was significantly elevated compared with that in the U251 cells. Meanwhile, the total Akt (t-Akt) and PI3K (t-PI3K) protein levels were not significantly altered in either cell line (Fig. 8).

**Fig. 9:**
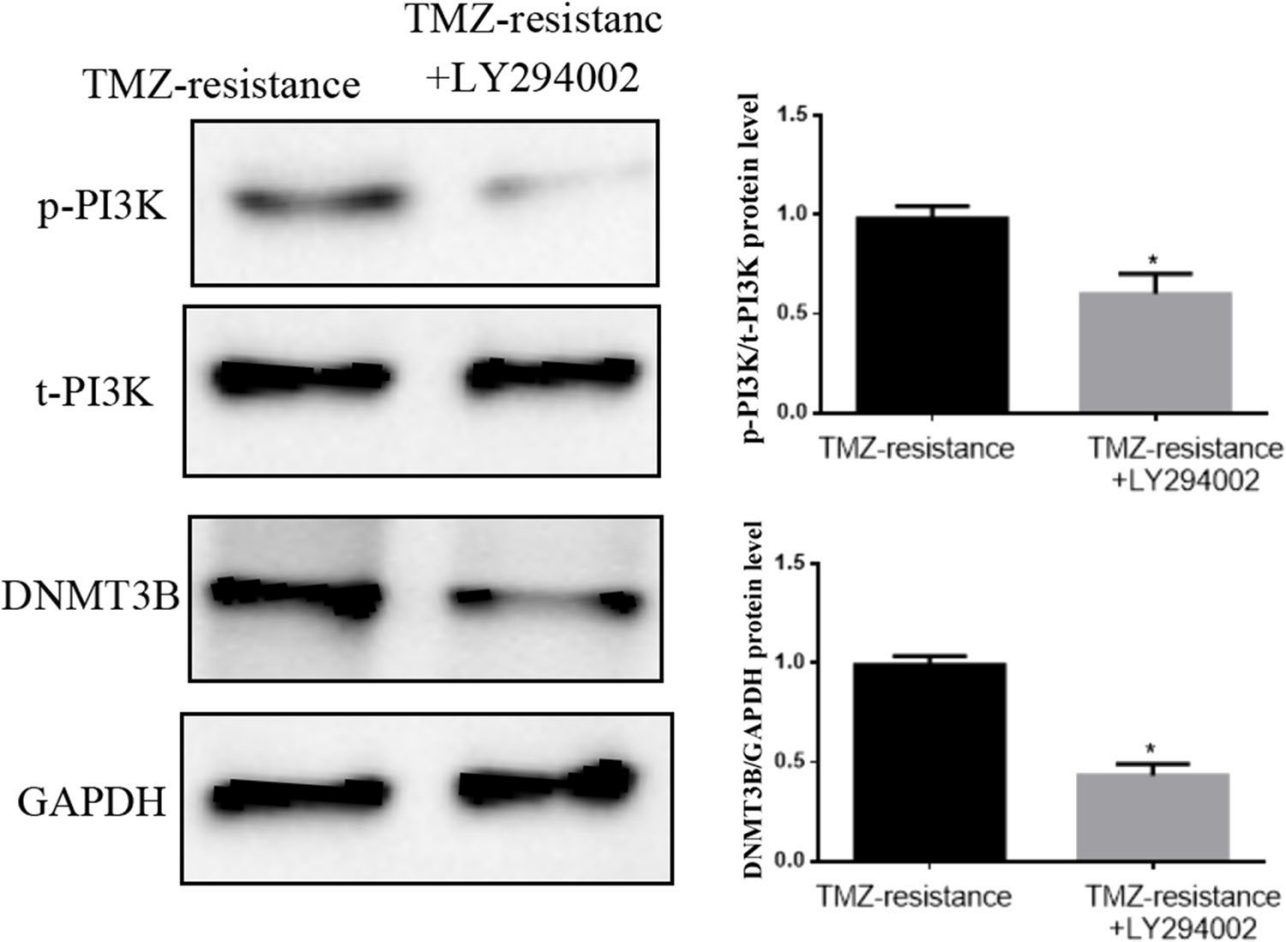
Western blot analysis showed that the p-PI3K and DNMT3B protein expression in U251-TMZ cells significantly increased compared with that in U251-TMZ cells treated with LY294002. *p < 0.05

The PI3K/Akt signaling pathway was pharmacologically inhibited with a specific PI3K inhibitor, LY294002, to verify whether the activated PI3K/Akt signaling pathway affects the chemoresistance of U251-TMZ cells by regulating DNMT3B expression. The results showed that LY294002 significantly inhibited PI3K phosphorylation and significantly reduced DNMT3B expression in the U251-TMZ cells (Fig. 9). These findings indicated that the PI3K/Akt pathway plays an important role in the chemosensitivity of GBM cells to TMZ by regulating DNMT3B expression.

This study revealed that DNMT3B expression levels were positively regulated by the PI3K/Akt signaling pathway in GBM, indicating that the activation of the PI3K/Akt signaling pathway may increase DNMT3B expression. The DNA methylation level tended to increase with the DNMT3B expression, and the chemosensitivity of U251 cells to TMZ increased with the DNA methylation level.

## Discussion

GBM is the most common primary malignant brain tumor. These tumors are highly infiltrative and proliferative, resulting in a median survival of 12–15 months after diagnosis. Patients often have a poor prognosis, causing an enormous burden on their families and the society. The poor prognosis is attributed to chemoresistance to TMZ, the first-line drug for treating GBM. Therefore, identifying an effective target for therapy is urgently needed. DNMT3B, which has de novo DNA methylation, plays an important role in the occurrence and development of tumors and the regulation of chemotherapy resistance [11, 16, 19]. Consistent with those reports, our study showed that DNMT3B mRNA expression was significantly upregulated in the U251-TMZ cell line compared with that in the U251 cell line. This result suggested that DNMT3B may play an important role in TMZ resistance in GBM cells. Further study showed that DNMT3B expression was significantly decreased in the U251-TMZ cell line transfected with DNMT3B-siRNA lentiviral vectors compared with that in the control group.

Our study aimed to clarify the role of DNMT3B in TMZ resistance in GBM cells and the mechanism by which the PI3K/Akt pathway mediates DNMT3B expression to influence the chemosensitivity of GBM to TMZ. We discovered that DNMT3B mRNA expression was significantly upregulated in the U251-TMZ cell line compared with that in the U251 cell line. The p-Akt and p-PI3K protein expression in the U251-TMZ cells was also significantly elevated. We then altered the protein expression of DNMT3B and suppressed the PI3K/Akt pathway to improve the chemosensitivity of the U251-TMZ cell line to TMZ. Research in recent decades has shown that aberrant DNA methylation is important in the promotion of tumor evolution and chemoresistance [11, 22].

We also found that DNMT3B downregulation inhibited proliferation and promoted chemosensitivity to TMZ in U251-TMZ cells in vitro and inactivated the PI3K/Akt signaling pathway. As a core factor of our study, the PI3K/Akt pathway regulates the expression of the downstream gene DNMT3B. DNMT3B mRNA expression has been recently associated with increased cancer aggressiveness [23]. Previous studies demonstrated that DNMT3B overexpression might be associated with resistance to chemotherapeutics in acute myeloid leukemia and prostate cancer cells [24, 25]. Mei [21] showed for the first time that the PI3K/Akt pathway regulates DNMT3B expression in hepatocarcinogenesis. This finding was consistent with the results of the present study. Our work demonstrated that suppressing the PI3K/Akt pathway by LY294002 can downregulate DNMT3B gene and low DNMT3B expression is closely related to chemosensitivity to TMZ in U251-TMZ cells. These data suggested that the DNMT3B protein is important for tumor deterioration and plays a crucial role in chemoresistance to TMZ in GBM.

DNA methylation is controlled by DNA methyltransferases, which utilize the methyl donor S-adenosyl methionine [21]. DNMT3B, a de novo methyltransferase, is highly expressed and frequently upregulated in many malignant tumors [19]. Gene amplification and protein overexpression of DNMT3B are associated with decreased sensitivity to decitabine and azacytidine in pancreatic and breast cancer cell lines [26]. In addition, DNMT3B depletion in DNMT3B-overexpressing colon cancer cell lines induced apoptosis and inhibited proliferation [27]. We observed that the mRNA expression of DNMT3B in GBM cells was inversely correlated with sensitivity to TMZ treatment in vitro. In addition, TMZ effectively induced apoptosis when DNMT3B expression was inhibited in our U251-TMZ cell lines.

Among epigenetic therapies, DNA methyltransferase inhibitors have been used for treating tumors, either individually or in combination [28]. We are curious as to whether DNMT3B mediates the hypermethylation of some downstream genes and promotes GBM chemoresistance to TMZ. Can concurrent treatment with DNA methyltransferase inhibitors increase the sensitivity of GBM to TMZ? These puzzling and fascinating aspects will be the focus of our next studies. We also need to evaluate the possible role of DNMT3B as a biomarker for TMZ treatment in GBM.

## Conclusion

DNMT3B facilitates resistance to TMZ in GBM. The PI3K/Akt pathway mediates DNMT3B mRNA expression, and its suppression can downregulate DNMT3B expression. Moreover, DNMT3B downregulation enhances the chemosensitivity and inhibits the malignant progression of GBM. Our findings provide new insights into the mechanism of TMZ resistance in GBM cells and identify DNMT3B as a promising therapeutic candidate target for GBM. However, the molecular mechanism underlying the enhanced chemosensitivity of GBM to TMZ induced by DNMT3B downregulation remains unknown. Our study has certain limitations. In the future, we will use DNMT3B knockout mice to explore the underlying mechanism of TMZ resistance in GBM cells.

## Data Availability

All data generated or analyzed in this study are available from the corresponding author upon reasonable request.
